# Scaling relationships of twig biomass allocation in *Pinus hwangshanensis* along an altitudinal gradient

**DOI:** 10.1371/journal.pone.0178344

**Published:** 2017-05-26

**Authors:** Man Li, Yuan Zheng, RuiRui Fan, QuanLin Zhong, DongLiang Cheng

**Affiliations:** 1 Fujian Provincial Key Laboratory of Plant Ecophysiology, Fujian Normal University, Fuzhou, Fujian Province, China; 2 Key Laboratory of Humid Subtropical Eco-geographical Process, Ministry of Education, Fuzhou, Fujian Province, China; Pacific Northwest National Laboratory, UNITED STATES

## Abstract

Understanding the response of biomass allocation in twigs (the terminal branches of current-year shoots) to environmental change is crucial for elucidating forest ecosystem carbon storage, carbon cycling, and plant life history strategies under a changing climate. On the basis of interspecies investigations of broad-leaved plants, previous studies have demonstrated that plants respond to environmental factors by allocating biomass in an allometric manner between support tissues (i.e., stems) and the leaf biomass of twigs, where the scaling exponent (i.e., slope of a log—log linear relationship, α) is constant, and the scaling constant (i.e., intercept of a log—log linear relationship, log β) varies with respect to environmental factors. However, little is known about whether the isometric scaling exponents of such biomass allocations remain invariant for single species, particularly conifers, at different altitudes and in different growing periods. In this study, we investigated how twig biomass allocation varies with elevation and period among *Pinus hwangshanensis* Hsia trees growing in the mountains of Southeast China. Specifically, we explored how twig stem mass, needle mass, and needle area varied throughout the growing period (early, mid-, late) and at three elevations in the Wuyi Mountains. Standardized major axis analysis was used to compare the scaling exponents and scaling constants between the biomass allocations of within-twig components. Scaling relationships between these traits differed with growing period and altitude gradient. During the different growing periods, there was an isometric scaling relationship, with a common slope of 1.0 (i.e., α ≈ 1.0), between needle mass and twig mass (the sum of the total needle mass and the stem mass), whereas there were allometric scaling relationships between the stem mass and twig mass and between the needle mass and stem mass of *P*. *hwangshanensis*. The scaling constants (log β) for needle mass vs. twig mass and for needle mass vs. stem mass increased progressively across the growing stages, whereas the scaling constants of stem mass vs. twig mass showed the opposite pattern. The scaling exponents (α) of needle area with respect to needle biomass increased significantly with growing period, changing from an allometric relationship (i.e., α < 1.0) during the early growing period to a nearly isometric relationship (i.e., α ≈ 1.0) during the late growing period. This change possibly reflects the functional adaptation of twigs in different growing periods to meet their specific reproductive or survival needs. At different points along the altitudinal gradient, the relationships among needle mass, twig mass, and stem mass were all isometric (i.e., α ≈ 1.0). Moreover, significant differences were found in scaling constants (log β) along the altitudinal gradient, such that species had a smaller stem biomass but a relatively larger needle mass at low altitude. In addition, the scaling exponents remained numerically invariant among all three altitudes, with a common slope of 0.8, suggesting that needle area failed to keep pace with the increasing needle mass at different altitudes. Our results indicated that the twig biomass allocation pattern was significantly influenced by altitude and growing period, which reflects the functional adaptation of twigs to meet their specific survival needs under different climatic conditions.

## Introduction

Biomass allocation to different organs (e.g., root, stem, and leaf) influences many aspects of plant growth and reproduction [1–2]. Given that biomass allocation is an important driving factor for the net carbon capture of plants and is sensitive to environmental change, research on the effects of environmental change on biomass allocation is crucial for understanding global carbon cycling, climate change, and plant life history strategies [3–4]. Considerable attention has been devoted to biomass allocation at different levels, e.g., the tree level [4–11], twig level [2–3, 12–15], and leaf level [16–19]. Furthermore, in many studies, the allometric function has been applied to describe plant biomass allocation: *M*_0_ = β*M*_1_^α^ (or log *M*_0_ = log β + α log *M*_1_), where *M*_0_ and *M*_1_ are different parts of plant biomass, α is the scaling exponent, and log β is the scaling constant. The scaling exponent (i.e., α < 1.0, ≈ 1.0, or α > 1.0) reflects different strategies of resource allocation in plants [20], and variation in the scaling constant along environmental gradients indicates how plants cope with environmental constraints [21].

The twig is an independent unit of plant growth and is the most active part of the plant branching system [12–13]. As leaves are directly involved in photosynthetic carbon gain, leaf traits (i.e., leaf area and biomass) are most sensitive to global change [22]. Thus, the biomass allocation pattern between the leaves and stems of twigs reflects not only the biochemical and hydraulic architecture characteristics of leaves [13, 23–24] but also plant configuration in specific environments [25–26]. It is well known that large-leaved or thick-stemmed twigs tend to have greater leaf extension and also larger leaf biomass [3, 12, 27]. Moreover, large-leaved species tend to have thick twigs, and thus, often need to increase the biomass allocation to support structures [16]. Theoretically, the scaling relationship between leaf and stem biomass in twigs can be deduced from the plant biomass allocation pattern at an individual level. Specifically, it generally indicates that leaf biomass scales isometrically with stem biomass in small plants lacking substantial quantities of secondary tissues (i.e., α ≈ 1.0) and scales less than one with stem biomass in larger plants (i.e., α < 1.0) [5, 7]. The reason for this decrease in scaling exponents with plant growth may be that there is a physiological limitation to the biomass allocation between photosynthetic and non-photosynthetic organs. In tree seedlings, almost all of the stem cross-section is physiologically active sapwood, whereas in older trees, only a small fraction is sapwood [28]. Thus, the scaling exponent must logically decrease during tree maturation. Furthermore, Niklas and Enquist suggested that annual growth rates of the leaves, stems, and roots of plants scale isometrically with each other [29]. Taken together, it is reasonable to assume that such isometric relationships between leaf and stem biomass at an individual level hold true within twigs, which represent the current-year growth of terminal branches in woody plants and may comprise mainly sapwood.

Indeed, some studies have suggested that leaf mass scales isometrically with stem mass in twigs, and that such an allocation pattern is not significantly affected by twig size but is greatly modulated by altitude [2]. For example, using 59 boreal woody species, Sun *et al*. found that the scaling relationship between the leaf and stem biomass responded to environmental gradients by changing the scaling constants, but that the scaling exponent held constant at the twig level [13]. However, whether these isometric scaling relationships hold true for single species, particularly conifers, remains unclear because interspecific biomass allocation patterns can often differ significantly from those at the intraspecific level [5, 30].

The scaling relationship of biomass allocation is sensitive to plant functional types, habitats, and species [12–13, 27, 31–35]. For example, Yang *et al*. reported that along a habitat gradient, the twig size—leaf size relationship differed both inter- and intraspecifically [21]. Cheng *et al*. also suggested that relationships of leaf mass with respect to leaf area differed significantly between inter- and intraspecific levels [36]. Moreover, there is a substantial body of evidence indicating that conifers and broad-leaved species differ in a variety of vegetative traits (e.g., leaf lifespan, conduit diameter, and growth rates) that might influence the biomass allocation patterns [37]. Indeed, comparative studies have indicated that the growth rates of conifers are generally slower than those of angiosperm species, at least during the juvenile phase of their development [38–39]. For example, Milla showed that the scaling exponents of leaf size and number differed between evergreen and deciduous species [34]. Additionally, Guo *et al*. indicated that the larger twigs of conifers allocated more biomass to the stem than to the needles [40]. Therefore, further studies are needed to clarify whether twig biomass allocation is consistent in coniferous and broad-leaved species. However, to the best of our knowledge, there have been no previous studies that have investigated how growing periods and environmental gradients affect twig biomass allocation in conifers.

Leaf area and leaf mass are crucial for plant metabolic performance, and many theoretical models have implied that the scaling exponent of leaf area vs. dry mass should be close to unity (i.e., α = 1.0), irrespective of variations in environmental conditions [13, 41–42]. However, the “diminishing returns” hypothesis proposed by Niklas *et al*. predicts that leaf area would scale less than one with respect to leaf dry mass at the individual leaf level (i.e., α< 1.0), indicating that the cost of light interception increases with an increase in leaf area [43–45]. Importantly, the “diminishing returns” hypothesis may also lead to the prediction that an allometric scaling relationship should hold true for the total leaf area and leaf biomass within twigs. Further, some studies have also suggested that the scaling exponent of leaf area vs. leaf mass increases disproportionately with increasing leaf mass (i.e., α> 1.0) [46–47]. Therefore, it remains unclear whether and how the relationship between leaf area and leaf mass within twigs varies among species under different environmental conditions and growing periods.

In this study, we empirically determined how the twig biomass allocation pattern in *Pinus hwangshanensis* Hsia trees growing along an altitudinal gradient on Wuyi Mountain was affected by different environments and stages of the growing period. *P*. *hwangshanensis*, a tree species endemic to China, is a constructive species of representative formation, and is also an important afforestation tree species in the central mountain regions of the subtropics [48]. Previous studies on *P*. *hwangshanensis* have primarily focused on how tree growth responds to environmental conditions such as soil nutrients or climate [49]. However, the relationship between the biomass distribution of stems and leaves of twigs along an altitudinal gradient at different stages of the growing period remains unclear. Furthermore, altitudinal gradients are considered as powerful natural laboratories for examining the ecological responses of plants to geophysical influences [50]. Thus, our data will provide a unique opportunity to evaluate how biomass allocation patterns in the twigs of conifers respond to environmental changes. Specifically, the objectives of this study were to addresses the following issues: (i) determine whether needle size is independent of twig size in *P*. *hwangshanensis*; (ii) determine whether habitats influence the biomass allocation between leaf and stem within a twig; and (iii) examine the scaling relationship for leaf area with respect to leaf mass at different altitudes and in different growing periods.

## Materials and methods

### Study site

With the permission of the Administrative Bureau of Jiangxi Wuyishan National Nature Reserve, the experiment was conducted in the Wuyishan National Nature Reserve, Jiangxi Province, southeastern China (27°48′11″–28°00′35″N, 117°39′30″–117°55′47″E). The reserve has an elevation varying from 350 to 2160.8 m above sea level (a.s.l.) and a mid-subtropical monsoon climate. Mean annual precipitation is approx. 2050 mm at low altitude, 2530 mm at medium altitude, and 2820 mm at high altitude [51]. The annual mean temperature decreases from 13°C at 1200 m a.s.l. to 11°C at 1600 m a.s.l. and 9°C at 2000 m a.s.l. (with the maximum and minimum temperatures occurring in July and January, respectively). The soil texture is mainly a sandy clay loam [52].

To examine the pattern of biomass allocation along an altitude gradient, three sample sites at low, medium, and high altitudes, were established in the nature reserve. Nine plots were sampled in total, with three being located at low altitude (1200 m a.s.l.), three at medium altitude (1600 m a.s.l.), and three at high altitude (2000 m a.s.l.). Each plot (20 m × 20 m) was located at least 20 m from the stand edge. Forest canopy closure, stand density, stem diameter at breast height (DBH), and plant height (H) were measured within each plot. The sample information is shown in Table 1.

**Table 1:** Description of *Pinus hwangshanensis* plots at different altitudes (mean ± *SE*).

Altitude (m)	Forest canopy closure (%)	Stand density (trees/hm^2^)	Mean DBH (cm)	Mean height (m)	LMA (mg/mm^2^)
L(1200m)	0.9 ± 0.012a	3160 ±240a	23.99 ± 0.79a	16.19 ± 0.46a	0.11 ± 0.003b
M(1600m)	0.82 ± 0.044a	2460 ±380a	14.53 ± 0.40b	10.49 ± 0.16b	0.11 ± 0.002b
H(2000m)	0.49 ± 0.031b	930 ±150b	13.17 ± 0.66b	4.76 ± 0.14c	0.10 ± 0.003a

L, low altitude; M, middle altitude; H, high altitude; LMA, leaf mass per area

Different letters in a column indicate that significant differences exist between altitudes (*P*<0.05).

### Plant sampling

Twigs have been defined as the terminal branches of current-year shoots, consisting of stems, laminas, and petioles [2, 13, 15]. In the present study, the twigs of *P*. *hwangshanensis* consisted of needles (without petioles) and stems, and were always unbranched. In December (late growing period, LG) of 2014 and June (early growing period, EG) and September (mid-growing period, MG) of 2015, 45 mature individuals were randomly selected at each of the three altitudes. For each tree, five branches with tips at the outer edge of the plant’s crown were randomly selected to avoid growth differences resulting from varying light conditions. From each selected branch (without apparent leaf area loss), fully mature needles were collected for measurement. Twig length and needle number were recorded for each twig. Needle areas were scanned with an EPSON Perfection v37, and the projection area of needles was digitized using Photoshop software (because the needles have a certain three-dimensional structure, the needle area was the projection area and not the actual area of the needle; however, we ignored this factor here). The samples were oven-dried to constant mass and weighed. Stem mass, needle mass, twig mass, and leaf mass per unit area were measured separately for each twig. The twig mass was the sum of the total needle mass and the stem mass, and leaf mass per area was calculated as the ratio of the individual needle mass and the individual needle area.

### Data analysis

The values for the twig and leaf traits were arithmetically averaged, and the trait averages were then log_10_-transformed to generate a normal distribution. Model Type II regression was used to estimate the scaling exponents and scaling constants using the (Standardized) Major Axis Estimation package “smatr” version 3.4–3 in R software [53–54]. The package was also used to provide the Model Type II equivalent of OLS standard analyses of covariance [53, 55]. The significance level for testing slope heterogeneity was *P* < 0.05 (e.g., slope heterogeneity was rejected if *P* > 0.05). The data for altitudes and periods showing no statistically significant differences in the numerical values of α were examined to determine common scaling exponents using the standardized major axis package in R [53, 55]. For several bivariate groups of observations, this function was used to determine whether the line-of-best-fit had a common slope for all samples, when the line-of-best-fit was estimated using the major axis, standardized major axis, or a more general version of these methods in which the error variance ratio was estimated from the data.

## Results

### Biomass allocation of twigs during different stages of growing periods

**Fig 1:**
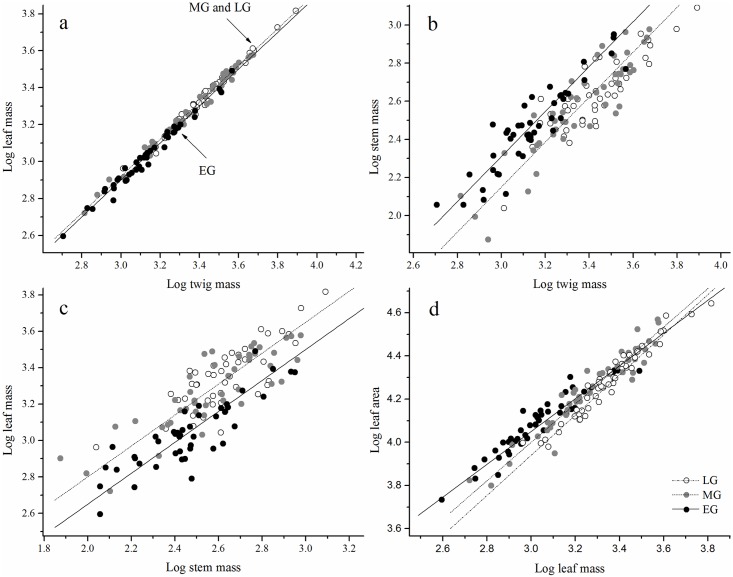
The scaling relationships between twig biomass allocations of *Pinus hwangshanensis* in different periods. The circle symbol indicates late growing period (LG), the gray symbol indicates mid-growing period (MG), and the black symbol indicates early growing period (EG).

Needle mass scaled nearly isometrically with twig mass in the three different stages of the growing period (i.e., α ≈ 1.0, *P* < 0.001) with a common slope of 1.0 (95% CI = 0.98–1.02, *P* = 0.38) (Table 2 and Fig 1a). However, the scaling constants (log β) of needle vs. twig biomass in the mid- and late growing periods were significantly higher than that in the early growing period (Table 2 and Fig 1a), suggesting that more needle biomass was supported by a given twig mass in the late growing period.

**Table 2:** Relationships between biomass allocations at different stages of the growing period.

*y-x*	Growth period	n	*r*^2^	*P*	Scaling exponent (α)	95% CI of α	Scaling constant (log β)
*NM-TM*	EG	45	0.982	< 0.001	0.99	0.95–1.03	-0.07
	MG	45	0.989	< 0.001	0.99	0.96–1.02	-0.05
	LG	45	0.984	< 0.001	1.02	0.98–1.06	-0.16
*SM-TM*	EG	45	0.840	< 0.001	1.18	1.05–1.34	-1.25
	MG	45	0.818	< 0.001	1.20	1.05–1.37	-1.47
	LG	45	0.720	< 0.001	1.13	0.96–1.32	-1.22
*NM-SM*	EG	45	0.733	< 0.001	0.83	0.71–0.98	0.98
	MG	45	0.732	< 0.001	0.82	0.70–0.96	1.16
	LG	45	0.602	< 0.001	0.91	0.75–1.10	0.95
*NA-NM*	EG	45	0.902	< 0.001	0.76	0.69–0.84	1.77
	MG	45	0.919	< 0.001	0.89	0.82–0.97	1.33
	LG	45	0.948	< 0.001	0.93	0.87–1.00	1.15

Stem biomass scaled allometrically (i.e., α > 1.0) with twig biomass in the early and mid-growing periods, and differed significantly from 1.0 (*P* < 0.001, Table 2 and Fig 1b). However, the scaling exponents (α) of stem vs. twig biomass in the late growing period did not differ significantly from 1.0 (i.e., α = 1.13, 95% CI = 0.96–1.32) (Table 2 and Fig 1b). Despite this variation, the scaling exponents (α) of stem vs. twig biomass were indistinguishable for the three growing periods, with a common slope of 1.18 (95% CI = 1.09–1.27, *P* = 0.81) (Table 2 and Fig 1b). However, the scaling constant (log β) of stem vs. twig biomass in the early growing period was higher than that in the mid- and late growing periods (Fig 1b).

The scaling relationship between needle and stem biomass showed the opposite pattern to the scaling relationship between the stem and twig biomass. Specifically, the needle biomass scaled allometrically with stem biomass in the early and mid-growing periods (i.e., α = 0.83 and 0.82 for early and mid-growing period, respectively), whereas needle biomass scaled nearly isometrically with stem biomass in the late growing period (i.e., α = 0.91, 95% CI = 0.75–1.10) (Table 2 and Fig 1c). Overall, the scaling exponents (α) of needle vs. stem biomass were indistinguishable among the three stages of the growing period, with a common slope of 0.85 (95% CI = 0.77–0.93, *P* = 0.72) (Table 2 and Fig 1c). The scaling constant (log β) of needle vs. stem biomass in the early growing period was significantly lower than that in the late growing period (Fig 1c).

Needle area was significantly correlated with needle mass in all stages of the growing period (all *P* < 0.001; Table 2). The values of the scaling exponent (α) were 0.76 (95% CI = 0.69–0.84), 0.89 (95% CI = 0.82–0.97), and 0.93 (95% CI = 0.87–1.00) for the early, mid-, and late growing periods, respectively (Table 2 and Fig 1d), suggesting that the relationship between needle area and needle biomass shifted from an allometric relationship (i.e., α < 1.0) in the early growing period to a nearly isometric relationship (i.e., α ≈ 1.0) in the late growing period.

### Biomass allocation in twigs at different altitudes

**Fig 2:**
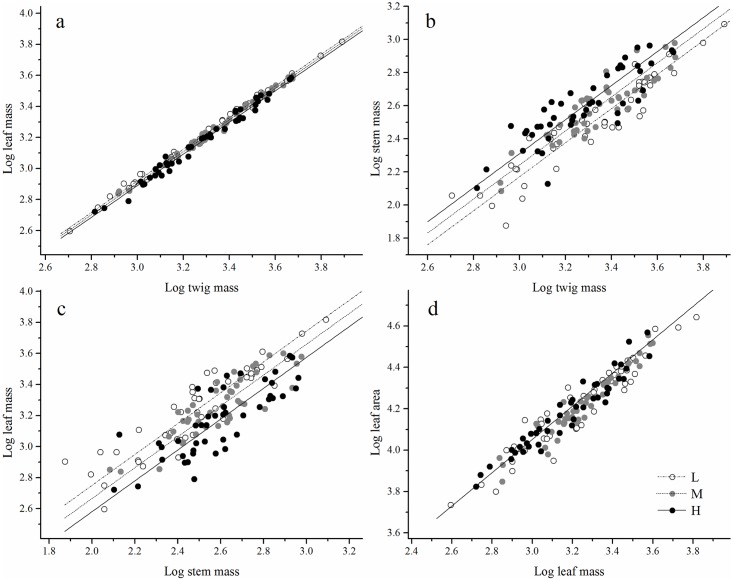
The scaling relationships between twig biomass allocations of *Pinus hwangshanensis* along an altitudinal gradient. The circle symbol indicates low altitude (L), the gray symbol indicates medium altitude (M), and the black symbol indicates high altitude (H).

Needle mass scaled nearly isometrically (i.e., α ≈ 1.0) with twig mass at the three studied altitudes (*P* < 0.001), with a common slope of 1.02 (95% CI = 1.00–1.03, *P* = 0.38) (Table 3 and Fig 2a). However, the scaling constants (log β) of needle vs. twig biomass were lower at the high altitude than at medium and low altitudes (all *P* < 0.001) (Fig 2a), suggesting that a relatively greater stem biomass was supported at high altitude.

**Table 3:** Relationships of twig biomass allocations at three altitudes.

*y-x*	Altitude	n	*r*^*2*^	*P*	Scaling Exponent (α)	95% CI of α	Scaling constant (log β)
*NM-TM*	L	45	0.996	< 0.001	1.01	0.99–1.03	-0.12
	M	45	0.988	< 0.001	1.01	0.98–1.05	-0.13
	H	45	0.985	< 0.001	1.04	1.01–1.08	-0.25
*SM-TM*	L	45	0.875	< 0.001	1.02	0.91–1.13	-0.88
	M	45	0.813	< 0.001	1.06	0.93–1.21	-0.95
	H	45	0.746	< 0.001	1.01	0.86–1.17	-0.70
*NM-SM*	L	45	0.830	< 0.001	1.0	0.88–1.13	0.75
	M	45	0.724	< 0.001	0.95	0.81–1.12	0.78
	H	45	0.636	< 0.001	1.04	0.86–1.25	0.48
*NA-NM*	L	45	0.908	< 0.001	0.79	0.72–0.87	1.65
	M	45	0.930	< 0.001	0.84	0.78–0.91	1.49
	H	45	0.926	< 0.001	0.78	0.72–0.85	1.71

TM, twig mass; NM, needle mass; SM, stem mass; NA, needle area; L, low altitude; M, middle altitude; H, high altitude.

Stem mass scaled nearly isometrically (i.e., α ≈ 1.0) with twig mass at all three altitudes, with a common slope of 1.03 (95% CI = 0.96–1.11, *P* = 0.83) (Table 3 and Fig 2b). However, the scaling constant (log β) of stem vs. twig biomass was higher at the high altitude than at the medium and low altitudes, suggesting that more leaf biomass was supported by a given twig mass at lower altitudes (all *P* < 0.001) (Fig 2b).

Needle biomass scaled isometrically (i.e., α ≈ 1.0) with stem biomass, with a common slope of 0.99 (95% CI = 0.91–1.08, *P* = 0.78) (Table 3 and Fig 2c). Moreover, a significant difference in the scaling constants (log β) for needle mass vs. stem mass was found among the three altitudes (all *P* < 0.001) (Table 2 and Fig 2c).

Needle area scaled allometrically (i.e., α < 1.0) with leaf biomass at all three altitudes, (*P* < 0.001), with a common slope of 0.8 (95% CI = 0.77–0.85, *P* = 0.38) (Table 3 and Fig 2d), suggesting that needle area failed to keep pace with the increasing leaf mass at different altitudes. Moreover, there was no difference in the scaling constants (log β) among the three altitudes, with a common constant of 1.64 (95% CI = 1.52–1.77, *P* = 0.05) (Table 3 and Fig 2d).

## Discussion

### The scaling relationships between needle area and needle biomass

The scaling relationship between leaf size and twig size reflects the outcome of an evolutionary trade-off among many ancestral metabolic, morphological, and anatomical traits, which has important implications for understanding the ability of plants to acclimate to environmental conditions [43, 56]. West *et al*. [41–42] assumed that the scaling exponent of leaf area and leaf biomass was close to 1.0. However, our data revealed that the scaling exponents of needle area vs. needle biomass were less than 1.0 (i.e., α = 0.76) in the early growing period and shifted to nearly 1.0 (i.e., α = 0.93) in the late growing period (Table 2 and Fig 1d), indicating that the allometric exponents between needle area and needle biomass were closely related to the stages of the plant growing period. Similarly, previous studies have indicated that metabolic rates differed among different stages of growing periods [57–58].

Furthermore, needle area scaled allometrically (i.e., α < 1.0) with needle mass at different altitudes (Table 3 and Fig 2d). These results contrast with those reported by Pan *et al*., who showed that the scaling exponent of leaf area with respect to biomass varied from less than 1.0 at low altitudes to more than 1.0 at high altitudes [47]. Moreover, our results are inconsistent with those of Luo *et al*., who demonstrated that below a critical altitude, specific leaf area (SLA) increased significantly with increasing altitude, whereas above the critical altitude, SLA showed the opposite pattern [59]. Our results at the individual leaf level are, however, consistent with the predictions of the “diminishing returns hypothesis” proposed by Niklas *et al*. [43, 44]. Specifically, the diminishing returns hypothesis proposes that an increasing leaf area would fail to keep pace with increasing leaf biomass and lead to an allometric scaling relationship between leaf area and leaf biomass (i.e., α < 1.0) [44].

The scaling of leaf area vs. leaf mass affects leaf economy in size-dependent ways, which has an important implication for leaf size optimization [13]. In the present study, needle area scaled disproportionately slower than needle mass, and smaller needles showed higher SLA than larger needles at different altitudes (Table 3 and Fig 2d). SLA reflects a resource allocation strategy within the individual leaf [23], and thus reacts strongly when changes in internal or external cues are perceived by the plant. Consequently, SLA decreases with increasing altitude and growth period length, and in old leaves and plants, which is understood to reflect an adjustment to temperature and internal conditions [60]. In addition, extensive heterogeneity in the leaf size dependency of environmental and developmental effects on SLA can develop, depending on whether changes in SLA are predominantly due to thickness or density variation, and whether the support needs of increasing leaf size are met by increasing support within the leaf by stronger petioles or by increasing branch level resistance [34].

### The scaling relationships between needle and twig biomass

The scaling relationships among the biomasses of different plant organs have been demonstrated in numerous experimental studies from the individual to the community level [35, 61]. Our results showed that needle biomass scaled isometrically to twig biomass at different stages of the growing period and at different altitudes (Figs 1a and 2a), indicating that environmental factors and growing periods had no significant effect on the scaling exponents of needle biomass with respect to twig biomass in *P*. *hwangshanensis*. Such an isometric pattern of needle vs. twig biomass is consistent with the results of Xiang *et al*. and Yang *et al*., who demonstrated that the biomass allocation for small plants at the individual level might be applicable at the twig level [2, 15]. On the basis of the assumption of a fractal network structure in plants, West *et al*. predicted that leaf biomass scaled as the 3/4 power of stem biomass, root biomass, and total biomass for larger plants [41–42]. Subsequently, empirical studies and metabolic scaling theory (MST) have also indicated that leaf biomass scales nearly one to one with stem and total biomass for individual plants [26, 35].

However, the findings of our study are apparently contrary to the results of Pickup *et al*. and Niinemets *et al*., who showed that needle biomass scaled allometrically with twig biomass (i.e., α < 1.0) [3, 16]. This discrepancy may have two sources. One source of discrepancy is related to the sampling method. Pickup *et al*. studied the relationships of twig biomass allocation using a cross-sectional area of more than 10 mm^2^ in 70 woody species [3]. In the present study, we collected terminal young branches, which can more accurately reflect the cost—benefit tradeoffs of the plant [2]. The second source is related to the research level and environmental conditions. Pickup *et al*. carried out their study at the inter-specific level under conditions of high precipitation and high nutrient availability [3]. Previous studies have indicated that the distribution patterns of biomass are associated with nutrients, light, water, temperature, plant growth form, stand structure, stand age, habitat, climate, and other environmental factors [8–9, 26, 62–64].

In the present study, needle mass scaled nearly isometrically with twig mass (i.e., α ≈ 1.0) (Table 3 and Fig 2a), indicating that twig size does not significantly affect needle biomass allocation. That is, thick-twigged or large-leaved species do not have a higher proportion of needle biomass allocation relative to the small ones in *P*. *hwangshanensis*. However, large twigs may enhance plant competitive potential because they minimize branching, thereby facilitating sapling growth into the unshaded overstory. Moreover, although large twigs require large biomass and carbon investment, sometimes, carbon is not a suitable estimator of twig construction costs when the supply of mineral nutrients, rather than carbon, limits growth.

### Response of twig biomass allocation to altitude and growing period

Biomass allocation provides a basis for species evolution, plant life history strategy, and carbon cycling, and is key to understanding carbon partitioning and carbon sequestration functions in ecosystems [65–66] Biomass allocation is related to many other plant characteristics, such as leaf area index, litter decomposition, nutrient and water uptake rates, carbon turnover, and species composition. Furthermore, it has a profound influence on life history strategies, evolutionary strategies, community structure, and terrestrial biogeochemical cycles [8, 26, 67–68]. Therefore, study of the biomass allocation of twigs is essential for an understanding of carbon storage and turnover and plant life history under different climate and habitat conditions.

Optimal allocation theory predicts that plants should invest more biomass among various plant organs that acquire the most limiting resource, to enable adaptation to environmental changes [69]. Biomass allocation patterns might reflect a self-coordinating mechanism of plants that maximizes resource utilization. In the present study, the scaling constants of needle vs. stem biomass in twigs of *P*. *hwangshanensis* were higher at lower altitude (Fig 2c). In other words, individuals at low altitude had a smaller stem mass, but a relatively larger needle mass than plants at medium and high altitudes, suggesting that *P*. *hwangshanensis* tends to have greater needle biomass allocation at low altitude and greater stem biomass allocation at high altitude.

Light, water, and temperature are important factors that affect biomass allocation patterns and community structure and function in mountain environments. At low altitude, the community coverage, density, and mean tree height of *P*. *hwangshanensis* are greater than those at medium and high altitudes (Table 1), and there is also greater interference from other adjacent species. *P*. *hwangshanensis* was shown to increase investment in needle biomass (Table 3 and Fig 2c), which maximizes photosynthetic efficiency. Previous studies have indicated that hydrothermal conditions are relatively advantageous for plant performance at low and medium altitudes, whereas vegetative growth is limited by low temperature at high altitude [70]. At high altitude, the temperature is generally low, which leads to a reduced tracheal diameter and a reduced efficiency in water and nutrient transport. However, maintaining a relatively constant hydraulic conductivity increases the resistance to freeze-induced xylem cavitation and embolisms [71–73]. Therefore, *P*. *hwangshanensi*s increases its input into the supporting components to meet the demands of nutrition and water transport at high altitude (Table 3 and Fig 2c).

In addition, Niklas reported that plants need to withstand the pressure created by strong winds and snowstorms at high altitude [74–75]. This is another reason why plants growing at high altitudes have more stringent requirements for physical support components. Moreover, previous studies have indicated that evergreen species growing at low altitude have longer leaf longevity and higher N content than those growing at high altitude. With increasing elevation along altitudinal gradients, subtropical evergreen species decrease in both diversity and abundance as the associated risks increase the need for both physical support and enhanced water and nutrient transport. Leaf mass per area (LMA) is smaller at high altitude than at medium and low altitudes, partly because the leaf mass and leaf area decrease with increasing altitude [76].

The scaling relationships between twig components and growing periods in plants have seldom been reported. Plants with unique life history strategies are considered to possess different biomass allocations due to differences in growth habits [77]. Periodal differences in biomass allocation should lead to different growth strategies in plants. In this way, biomass allocation is affected not only by environmental factors but also by period. In the present study, we observed that *P*. *hwangshanensi*s tended to allocate more biomass to needles in the mid- and late growing periods, and allocated more biomass to stems in the early growing period (Table 2 and Fig 1c). This growth pattern is primarily attributed to the fact that *P*. *hwangshanensis* begins to branch out in the early growing period, and subsequently increases investment in leaf biomass to improve performance in the mid- and late growing periods.

## Conclusions

In summary, allometric scaling relationships were detected both between stem mass and twig mass and between needle mass and stem mass, suggesting that plants tend to have more stem mass per unit support tissue in the early growing period than in the mid- and late growing periods. We detected an isometric scaling relationship between needle mass and twig mass at different stages of the growing period. At different altitudes, isometric relationships were found among needle mass, twig mass, and stem mass along an altitudinal gradient, indicating that twigs tend to allocate more biomass to needles at low altitude and more biomass to stems at high altitude. In addition, allometric scaling relationships between needle area and needle mass were found to have a common slope <1.0, suggesting that needle area failed to keep pace with the increase in needle size. This relationship might be associated with the capacity of needles to intercept sunlight.

## Supporting information

S1 Table Supporting information. (XLSX)
